# Antibiotic Treatment of Gram-Negative Bacterial Left Ventricular Assist Device Infections: A Systematic Review

**DOI:** 10.31083/RCM52885

**Published:** 2026-10-15

**Authors:** Parsa Jahangiri, Mehmet Ozgen, Laura Doornekamp, Kadir Caliskan, Nelianne Verkaik

**Affiliations:** ^1^Thoraxcenter, Department of Cardiology, Cardiovascular Institute, Erasmus MC University Medical Center Rotterdam, 3015 GD Rotterdam, Netherlands; ^2^Department of Medical Microbiology and Infectious Diseases, Erasmus MC University Medical Centre Rotterdam, 3015 GD Rotterdam, Netherlands

**Keywords:** left ventricular assist device, driveline infections, Gram-negative bacterial infections, *Pseudomonas aeruginosa*, antimicrobial treatment, protocolized treatment, heart-assist devices

## Abstract

**Background:**

Gram-negative bacterial left ventricular assist device (LVAD) infections pose significant challenges due to antimicrobial resistance and high relapse rates. Moreover, current standardized treatment guidelines remain limited. Thus, this study aimed to evaluate current antimicrobial strategies for Gram-negative bacterial LVAD infections, highlight treatment trends, and examine outcomes to inform clinical decision-making.

**Methods:**

A systematic review was conducted in accordance with the 2020 Preferred Reporting Items for Systematic Reviews and Meta-Analyses (PRISMA) guidelines. Studies were included if they reported antibiotic regimens for Gram-negative LVAD-specific infections and the clinical outcomes of these infections. Antibiotic regimens, associated management strategies, and clinical outcomes were summarized by infection location.

**Results:**

A total of 15 studies were included, reporting 25 cases of Gram-negative LVAD-specific infections and 28 identified Gram-negative pathogens. Most cases involved superficial or deep driveline/exit-site/pocket infections (16/25, 64.0%), whereas pump/cannula infections and device-specific bloodstream infections occurred in 3/25 (12.0%) and 6/25 (24.0%) patients, respectively. Antibiotic strategies varied widely in both selection and duration. Superficial driveline and exit-site infections were treated with a median of 14 days of antibiotics (interquartile range (IQR), 14–21.5; range, 7–49), whereas deep driveline/pocket infections required longer treatment with a median of 42 days (IQR, 31.5–42; range, 14–42). Pump/cannula and bloodstream infections often required prolonged therapy, suppressive treatment, and/or major surgical intervention.

**Conclusions:**

The treatment of Gram-negative bacterial LVAD infections remains highly variable and poorly characterized due to the limited number of reported cases, making it difficult to establish evidence-based clinical recommendations. This highlights the urgent need for further research to establish evidence-based treatment protocols and improve patient outcomes.

## 1. Introduction

Continuous-flow left ventricular assist devices (cf-LVADs) are increasingly used to provide cardiac support in patients with end-stage heart failure, serving both as bridge-to-transplant (BTT) and destination therapy (DT) [1,2]. However, complications including bleeding, external outflow graft stenosis, chronic kidney disease, and particularly infection remain major challenges [3,4,5,6]. Contemporary evidence suggests that LVAD infections affect approximately 19–39% of recipients [6].

The 2017 International Society for Heart and Lung Transplantation (ISHLT) consensus document, subsequently incorporated into the 2023 ISHLT Guidelines for Mechanical Circulatory Support, classifies infections in patients receiving mechanical circulatory support into three categories: MCS-specific infections, MCS-related infections, and non-MCS infections [7,8]. LVAD-specific infections are unique to LVAD recipients and involve components of the device, such as the driveline, pump pocket, and pump/cannula, or may present as device-specific bloodstream infections (BSI). LVAD-related infections, including mediastinitis and endocarditis, can also occur in non-LVAD patients but pose greater challenges in those with an LVAD. Non-LVAD infections, such as urinary tract infections, are unrelated to the presence of the device [7,8]. In the latest 2024 ISHLT infection definitions, LVAD-related and non-LVAD infections have been consolidated into a single category, termed non-MCS-specific infections, due to their distinct pathophysiology compared with LVAD-specific infections [9].

LVAD infections significantly impact patient outcomes and healthcare costs, often requiring prolonged hospitalization, extensive antimicrobial therapy, and invasive surgical interventions [6,10,11]. Deep device infection is particularly challenging and frequently requires surgical source control or transplantation [12].

Although gram-positive bacteria remain the most common pathogens in LVAD infections, Gram-negative bacteria, particularly *Pseudomonas aeruginosa*, are increasingly prevalent, accounting for 22–28% of LVAD-specific infections [6,8,13]. Moreover, Gram-negative pathogens are more frequently observed in late-onset LVAD infections [14]. Despite their growing clinical significance, current ISHLT guidelines provide limited recommendations for the treatment of Gram-negative LVAD infections. In the absence of robust clinical data, management often relies on expert opinion or extrapolation from other infectious disease guidelines [7,9]. While individualized care is essential, the ISHLT guideline authors acknowledge in the recent consensus statement that general treatment frameworks could provide valuable guidance to clinicians managing these infections.

This review therefore aims to evaluate current antimicrobial regimens used for Gram-negative LVAD-specific infections, assess their treatment success and clinical outcomes, and identify key gaps that future research should address.

## 2. Methods

### 2.1 Search Strategy and Screening

This systematic review was carried out according to the Preferred Reporting Items for Systematic Reviews and Meta-Analyses (PRISMA) statement. MEDLINE (Ovid), Embase, Cochrane CENTRAL, Google Scholar, and Web of Science were searched through April 6, 2026. Searches in MEDLINE, Embase, and Web of Science were limited to publications from 2005 onward, and the first 50 Google Scholar results were screened. The search strategies were developed by a biomedical information specialist with specific expertise in systematic review search strategies and combined controlled vocabulary and free-text terms related to left ventricular assist devices, including “LVAD”, “HeartMate”, and “HeartWare”, with terms related to Gram-negative bacteria and individual Gram-negative pathogens, including *Enterobacter*, *Klebsiella*, *Pseudomonas*, *Acinetobacter*, *Proteus*, *Serratia*, and *Escherichia coli*. The complete search strategy for each database is provided in **Supplementary Table 1**. The method used to translate search strings from one database to another has been described by Bramer et al. [15].

### 2.2 Eligibility Criteria and Study Selection

Two reviewers (PJ and MO) independently reviewed all search results. Disagreements were resolved by consultation with a third reviewer (LD). The retrieved citations were first assessed by title and abstract screening, after which full texts were evaluated using the predefined inclusion and exclusion criteria. Title and abstract screening was deliberately broad to maximize sensitivity. Records were retained if the title or abstract referred to LVAD patients and any potentially relevant infectious complication, or if eligibility remained unclear. We manually searched the reference lists of included studies to identify additional potentially eligible studies. Studies were included if they described antibiotic treatment, including treatment duration, route of administration, or antibiotic type, for adult LVAD patients with a first Gram-negative LVAD-specific infection and reported at least one predefined outcome measure. Studies were excluded if they focused on pediatric patients, reported polymicrobial infections involving both Gram-positive and Gram-negative pathogens, or were published in languages other than English.

### 2.3 Definitions and Data Extraction

The location of included LVAD-specific infections was classified according to ISHLT definitions [8]. LVAD-specific infections involve any part of the device, including the driveline, exit site, pump, pocket, or cannula [8]. Therefore, following the ISHLT classification, we structured our analysis accordingly and analyzed antibiotic treatment regimens separately for each infection location. Cases where more than one site of the LVAD was infected were grouped into the category according to the site that determined both the selection of antibiotic and the duration of the antibiotic treatment course.

The following information was extracted from the included reports: demographic data (sex, age, and country); clinical data (medical comorbidities, clinical presentation, LVAD type, LVAD goal, interval between LVAD implantation and infection, diagnostic tools including cultures and imaging findings, infected site, classification of LVAD infection, and causative microorganisms); antibiotic treatment regimen; and treatment-related management strategies and outcomes, including the need for suppressive antimicrobial therapy or surgical source control, infection-free period, relapse, reinfection, secondary infection, recurrent hospitalization, and death.

Definitions for relapse and reinfection were based on Verkaik et al. [16]. Relapse was defined as a second LVAD infection caused by the same species within six months after treatment initiation. Reinfection was defined as a second superficial or deep driveline infection, or BSI, caused by the same species more than six months after initial treatment. Secondary infection was defined as a new infection caused by a different species. Infection-related mortality was defined as mortality directly or indirectly caused by the primary infection, relapse, or reinfection.

### 2.4 Endpoints and Synthesis Methods

The variables of interest were the antibiotic regimens used and their associated treatment-related management strategies and outcomes for each LVAD-specific infection location. These included the need for suppressive antimicrobial therapy or surgical source control, the type of surgical intervention performed, relapse, reinfection, recurrent hospitalization, and infection-related mortality. Data were extracted from the manuscripts whenever available. Continuous variables are reported as median with interquartile range and range (minimum–maximum), Categorical variables are presented as numbers and percentages, n/N (%). All time-related data were converted to days.

## 3. Results

### 3.1 Literature Search and Study Characteristics

A flowchart of the systematic literature search is shown in Fig. 1. The search identified 687 papers, of which 437 remained eligible after duplicate removal. Following title and abstract screening, 289 papers were excluded. A total of 148 full-text reports were assessed for eligibility, but three were unavailable in full text, seven were not published in English, and 123 did not meet the inclusion criteria. Ultimately, 15 articles published between 2005 and 2026 met the inclusion criteria. The key characteristics of each included study are shown in Table 1 (Ref. [16,17,18,19,20,21,22,23,24,25,26,27,28,29,30]).

**Fig. 1.**
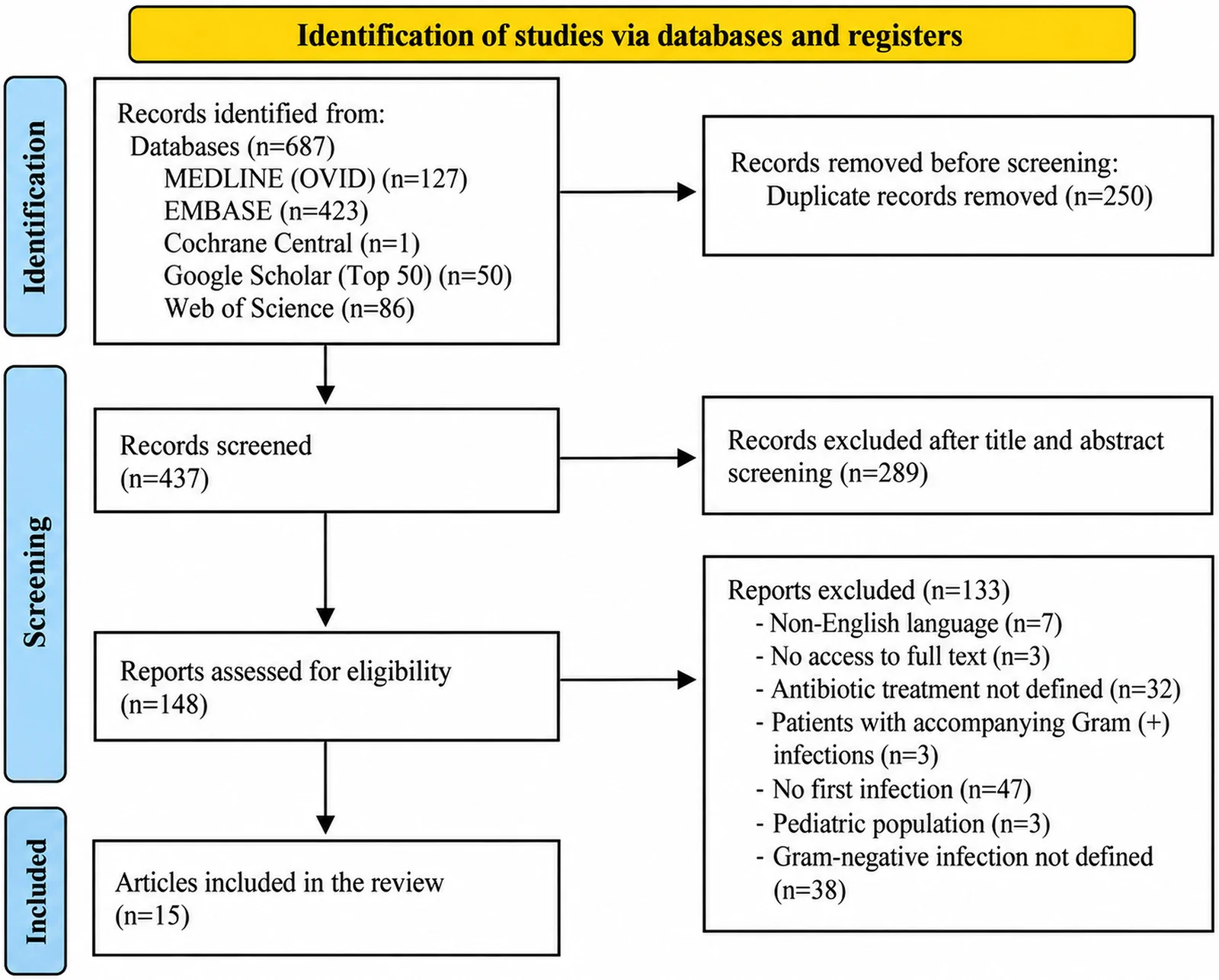
**Systematic review flow diagram**. The PRISMA flow diagram for the systematic review detailing the database searches, the number of abstracts screened and the full texts retrieved.

**Table 1.** **Baseline characteristics of all included cases**.

Reference	Country	Sex, age	Medical history and comorbidities	Clinical presentation	LVAD type	Goal	Duration from LVAD implantation to infection (days)
Simon et al. 2005 [17]	USA	Unknown	Unknown	Unknown	HeartMate I	BTT	Unknown
Manahan et al. 2006 [18]	USA	M, 66	Severe cardiac dysfunction	Wound dehiscence with exposed mesh, purulent drainage from wound, with surrounding cellulitis directly after LVAD implantation	Unknown	Unknown	0 days
		M, 53	Multi-vessel coronary artery disease and decreased EF, exacerbation of HF after a coronary artery bypass, RVAD	Symptomatic mediastinitis and wound dehiscence	Unknown	Unknown	30 days
Jiritano et al. 2013 [19]	Italy	M, 49	End-stage heart failure, renal failure requiring hemodialysis	The skin around the exit site of the abdominal cable became erythematous with serum and purulent discharge	HeartWare	Unknown	7 days
Bartoli et al. 2013 [20]	USA	M, 51	Non-ischemic cardiomyopathy	Fracture of the driveline at the insertion site on the pump	HeartMate II	BTT	487 days
Hill et al. 2014 [21]	USA	M, 64	Ischemic cardiomyopathy	Fever	HeartMate II	BTT	91 days
Formica et al. 2014 [22]	Italy	M, 56	Ischemic cardiomyopathy	Drainage and erythema around the driveline and local pain	HeartWare	Unknown	517 days
Levy et al. 2015 [23]	USA	M, 75	Unknown	Purulent discharge	HeartMate II	DT	547 days
Heil et al. 2015 [24]	USA	F, 25	Dilated cardiomyopathy, complicated VAD implantation (acute kidney injury, respiratory failure, tracheostomy, recurrent bleeding, ventricular arrhythmias)	Unknown	HeartWare + CentriMag (RVAD)	BTT	113 days
Dulanto Chiang et al. 2016 [25]	USA	M, 60	Dilated cardiomyopathy	Driveline exit site drainage for 3 months	HeartWare	BTT	152 days
		F, 63	Ischemic cardiomyopathy, diabetes	Discharge from the driveline site	HeartWare	BTT	152 days
Peghin et al. 2018 [26]	Italy	M, 49	Congestive heart failure due to acute myocarditis	Fever lasting 3 days	HeartMate II	BTT	150 days
Radcliffe et al. 2020 [27]	USA	F, 46	Unknown	Unknown	HeartMate II	Unknown	42 days
		F, 64	Unknown	Unknown	HeartMate II	Unknown	134 days
		M, 38	Unknown	Unknown	HeartMate 3	Unknown	292 days
		M, 74	Unknown	Unknown	HeartMate 3	Unknown	594 days
		F, 77	Unknown	Unknown	HeartWare	Unknown	730 days
Bodro et al. 2021 [28]	Spain	M, 66	Refractory chronic heart failure, slow-growing lung adenocarcinoma	Purulent tracheobronchitis	HeartMate 3	DT	Unknown
Kumar et al. 2021 [29]	USA	M, 59	Sarcoidosis, systolic heart failure, CRT-D	Fever, cough, worsening lower extremity edema and abdominal girth one week prior to admission	HeartWare	Unknown	Unknown
Verkaik et al. 2022 [16]	Netherlands	n = 4 Age/sex N/A	Unknown	Unknown	HeartMate 3	Unknown	Unknown
Cholevas et al. 2026 [30]	Germany	M, 43	End-stage ischemic heart failure	Asymptomatic; CT-detected abscess around the LVAD during routine transplant listing assessment	N/A	N/A	~4015 days

Abbreviations: BTT, bridge to transplantation; CRT-D, cardiac resynchronization therapy defibrillator; CT, computed tomography; DT, destination therapy; EF, ejection fraction; LVAD, left ventricular assist device; RVAD, right ventricular assist device; USA, United States of America; M, Male; F, Female.

### 3.2 Baseline Characteristics

A total of 25 cases were included. Among the 19 patients with available age data, the median age was 59 years (IQR, 49–65; range, 25–77 years). Among cases with available data, 14/19 (73.7%) were male. A total of 28 Gram-negative pathogens were identified in 25 cases, including two polymicrobial Gram-negative infections. Of the 28 identified pathogens, 12 (42.9%) were Enterobacterales, 14 (50.0%) were non-fermenting Gram-negative bacteria, and 2 (7.1%) were classified as other Gram-negative pathogens. The most frequently isolated pathogen was *Pseudomonas aeruginosa* (11/28, 39.3%), followed by *Klebsiella* spp. (4/28, 14.3%). Three isolates were reported as multidrug-resistant (MDR): one *Achromobacter xylosoxidans*, one *Pseudomonas aeruginosa*, and one *Stenotrophomonas maltophilia*. Among studies reporting LVAD type, HeartWare (7/22, 31.8%) and HeartMate 3 (7/22, 31.8%) were the most common devices. Among cases with known implantation indication, 8/10 (80.0%) received an LVAD as a bridge to transplantation, whereas 2/10 (20.0%) received an LVAD as destination therapy. Data availability differed across the treatment and outcome variables reported in Table 2. The number of contributing cases and reasons for missingness are summarized in **Supplementary Table 2**. The overall pathogen distribution is shown in **Supplementary Fig. 1**.

**Table 2.** **The distribution of Gram-negative pathogens and outcomes among the subgroups of left ventricular assist device specific infections**.

Identified pathogens	All pathogens (n = 28)	Superficial driveline/exit site infections (n = 11)	Deep driveline/pocket infections (n = 8)	Pump/cannula infections (n = 3)	Bloodstream infections (n = 6)
Enterobacterales	12 (42.9)	8 (72.7)	4 (50.0)	–	–
*Serratia marcescens*	3 (10.7)	2 (18.2)	1 (12.5)	–	–
*Proteus mirabilis*	3 (10.7)	1 (9.1)	2 (25.0)	–	–
*Escherichia coli*	1 (3.6)	1 (9.1)	–	–	–
*Klebsiella pneumoniae*	4 (14.3)	3 (27.3)	1 (12.5)	–	–
*Enterobacter*	1 (3.6)	1 (9.1)	–	–	–
Non-fermenting Gram-negative bacteria	14 (50.0)	3 (27.3)	4 (50.0)	2 (66.7)	5 (83.3)
*Pseudomonas aeruginosa*	11 (39.3)	2 (18.2)	3 (37.5)	2 (66.7)	4 (66.7)*
*Acinetobacter*	1 (3.6)	–	1 (12.5)	–	–
*Stenotrophomonas maltophilia*	1 (3.6)	1 (9.1)*	–	–	–
*Achromobacter xylosoxidans*	1 (3.6)	–	–	–	1 (16.7)*
Other Gram-negative pathogens	2 (7.1)	–	–	1 (33.3)	1 (16.7)
*Cardiobacterium valvarum*	1 (3.6)	–	–	–	1 (16.7)
*Coxiella burnetii*	1 (3.6)	–	–	1 (33.3)	–
**Outcomes**	**All patients (n = 25)**	**Superficial driveline/exit site infections (n = 8)**	**Deep driveline/pocket infections (n = 8)**	**Pump/cannula infections (n = 3)**	**Bloodstream infections (n = 6)**
Antibiotic course, median days (IQR; range)	Not pooled	14 (14–21.5; 7–49)	42 (31.5–42; 14–42)	Unknown	42 (21–51; 14–67)
Surgical source control, n/N (%)	9/21 (42.9)	1/6 (16.7)	4/7 (57.1)	3/3 (100.0)	1/5 (20.0)
Suppressive therapy, n/N (%)	9/24 (37.5)	4/8 (50.0)	1/8 (12.5)	1/3 (33.3)	3/5 (60.0)
Relapse, n/N (%)	8/25 (32.0)	3/8 (37.5)	2/8 (25.0)	0/3 (0.0)	3/6 (50.0)
Reinfection, n/N (%)	2/23 (8.7)	0/8 (0.0)	1/7 (14.3)	0/3 (0.0)	1/5 (20.0)
All-cause mortality, n/N (%)	10/20 (50.0)	4/8 (50.0)	1/3 (33.3)	2/3 (66.7)	3/6 (50.0)
Infection-related mortality, n/N (%)	7/20 (35.0)	2/8 (25.0)	0/3 (0.0)	2/3 (66.7)	3/6 (50.0)

*Isolates reported as multidrug-resistant by the original authors: one *Achromobacter xylosoxidans*, one *Pseudomonas aeruginosa*, and one *Stenotrophomonas maltophilia*. Because two driveline infections were polymicrobial, 28 pathogens were identified in 25 cases. Categorical variables are presented as n/N (%). Continuous variables are presented as median (interquartile range [IQR]; range). Specific source-control procedures are detailed in Tables 3,4,5. Heart transplantation was classified as surgical source control only when explicitly performed for infection control. For the summary counts, suppressive therapy initiated only after a documented relapse was not classified as suppression following the index episode. Only cases with available data were included in each analysis.

### 3.3 Superficial Driveline Infections

Five studies reported eight cases of Gram-negative superficial driveline/exit-site infection, involving 11 identified pathogens because two infections were polymicrobial (Table 2). The most frequently identified pathogens were *Pseudomonas aeruginosa* (2/11, 18.2%), *Klebsiella pneumoniae* (3/11, 27.3%), and *Serratia* spp. (2/11, 18.2%). Suppressive therapy was administered in 4/8 cases (50.0%). Superficial driveline infections were treated with a median antibiotic course of 14 days (IQR, 14–21.5; range, 7–49 days), most commonly using third-generation cephalosporins, fluoroquinolones, or trimethoprim/sulfamethoxazole. Relapse occurred in 3/8 cases (37.5%), while reinfection did not occur (0/8, 0.0%). Among six cases with known infection-control status, one (16.7%) required surgical source control, consisting of driveline revision. Heart transplantation occurred during follow-up in three cases, but the indication for transplantation was not reported. The infection requiring driveline revision included MDR *Stenotrophomonas maltophilia*. In one case, surgical intervention was declined; the patient subsequently experienced relapses with drug-resistant Enterobacterales and died one year later. Infection-related mortality was 2/8 (25.0%) (Table 3, Ref. [19,22,23,25,27]).

**Table 3.** **Characteristics and outcomes of patients with superficial driveline/exit-site infections**.

Reference, number of patients	Diagnostics	Gram-negative micro-organism	Therapeutic antibiotics (for LVAD infection)	Suppressive therapy after index episode	Surgical source control and heart transplantation	Infection free period/follow-up duration from first infection (in days)	Relapse/reinfection/secondary infection	Death during follow-up (cause)
Jiritano et al. 2013 [19]	Unknown	*Escherichia coli*	Ciprofloxacin, IV, 7 days	No	No	29 (became infection free after topical application of autologous platelet-rich plasma for 7 days after antibiotic treatment until death)	No relapse, no reinfection, no secondary infection	Yes, 50th day after LVAD implantation (due to MOF: infection-related)
Formica et al. 2014 [22]	Swab culture and after antibiotics showed no improvement, a CT was performed	*Pseudomonas aeruginosa*	Ceftazidime and amikacin, IV, 14 days	No	No	274 (became infection free after 4 weeks of frozen-and-thawed allogeneic platelet gel after the stop of antibiotics until end of follow up)	No relapse, reinfection or secondary infection during 9 months FU	No
Levy et al. 2015 [23]	Abdomen/pelvic CT and Ga-67 SPECT-CT	*Serratia*, *Klebsiella* and *Enterobacter*	Brief course of conventional IV AB followed by oral AB	No after the index episode; initiated after relapse and continued until death	No (recommended, but the patient declined)	30	Relapses with *Serratia marcescens* and drug-resistant organisms. No reinfection or secondary infection	Yes, 365 days after start infection (Relapses of LVAD infection: infection-related)
Dulanto Chiang et al. 2016 [25]	Unknown	*Klebsiella pneumoniae* and *S. maltophilia*	Ceftriaxone followed by amoxicillin-clavulanate	No	Driveline revision performed for infection control; later heart transplantation (indication not reported)	578 (from initial infection to secondary infection)	No relapse or reinfection. Secondary infections with *Pseudomonas* species	No
Radcliffe et al. 2020 [27]	Unknown	*Klebsiella pneumoniae*	Ceftriaxone IV + ciprofloxacin IV, 7 days Ciprofloxacin PO, 7 days	391 days, cefuroxime, PO	No	Unknown	No relapse or reinfection. Secondary infection with probable *Proteus mirabilis* and later with *Serratia marcescens*	Yes (*Serratia marcescens* bacteremia: not infection-related)
Radcliffe et al. 2020 [27]	Unknown	*Proteus mirabilis*	Cephalexin, PO, 7 days Trimethoprim/sulfamethoxazole, PO, 7 days Amoxicillin-clavulanate, PO, 10 days	162 days, amoxicillin	No other surgical intervention; heart transplantation later performed (indication not reported)	Unknown	Relapse with *Proteus mirabilis*. No reinfections. Secondary infections unknown	No
Radcliffe et al. 2020 [27]	Unknown	*Pseudomonas aeruginosa*	Ceftazidime, IV + ciprofloxacin, IV, 2 days Ciprofloxacin, IV + piperacillin-tazobactam, IV, 3 days Ciprofloxacin, PO + ceftazidime, IV, 14 days Ciprofloxacin, PO + ceftazidime, IV, 30 days	116 days, ciprofloxacin	No other surgical intervention; heart transplantation later performed (indication not reported)	Unknown	No relapse or reinfection. Secondary infections unknown	No
Radcliffe et al. 2020 [27]	Unknown	*Serratia marcescens*	Trimethoprim/sulfamethoxazole, PO, 14 days	234 days, trimethoprim/sulfamethoxazole, PO	No	>154 (from initiation of suppressive therapy until relapse)	Relapse with *Serratia marcescens*, No reinfection. Secondary infection unknown	Yes (not infection-related)

Abbreviations: CT, computed tomography; FU, follow-up; HTx, heart transplantation; IV, intravenous; LVAD, left ventricular assist device; MDR, multidrug-resistant; MOF, multiorgan failure; PO, oral administration.

### 3.4 Deep Infections

#### 3.4.1 Deep Driveline/Pocket Infections

Among the seven cases with a reported treatment duration, deep driveline/pocket infections were treated for a median of 42 days (IQR, 31.5–42; range, 14–42 days) (Table 4, Ref. [16,17,18,20,25,27,30]). Suppressive therapy was administered in 1/8 cases (12.5%). Surgical source control was performed in 4/7 patients (57.1%) and consisted of one LVAD exchange, one LVAD explantation at heart transplantation, and surgical driveline relocation in two patients. Relapse occurred in 2/8 cases (25.0%), and reinfection occurred in 1/7 cases (14.3%).

**Table 4.** **Characteristics and outcomes of patients with deep infections (deep driveline/pocket and pump/cannula infection)**.

Reference	Diagnostics	Causative micro-organism	Therapeutic antibiotics (for LVAD infection)	Suppressive therapy afterwards	Surgical source control and heart transplantation	Infection free period/ follow-up duration from first infection (in days)	Relapse/reinfection/secondary infection	Death during follow-up (cause)
Deep driveline/pocket infections								
Simon et al. 2005 [17]	Culture of driveline exit site drainage and blood. Also culture of device pocket during surgical drainage	*Acinetobacter*	42 days	No	Unknown	Unknown	Relapse with *Acinetobacter* BSI. Reinfection and secondary infection unknown.	Unknown
Bartoli et al. 2013 [20]	Unknown	*Pseudomonas* species	Levofloxacin	No	LVAD exchange performed for infection control	304 (resolution of infection due to antibiotics + device exchange until reinfection)	No relapse. Reinfection of the driveline and blood stream with *P. aeruginosa* and later a reinfection of the LVAD-pocket with *Pseudomonas*. No secondary infection during FU.	No
Dulanto Chiang et al. 2016 [25]	Unknown	*Proteus mirabilis*	Trimethoprim-sulfamethoxazole, 14 days	No	Heart transplantation with LVAD explantation performed for infection control after persistent driveline-site discharge	Approximately 47–78 (resolution of infection until relapse)	Relapse with *Proteus mirabilis*. No reinfection or secondary infection before HTx.	No
Radcliffe et al. 2020 [27]	Unknown	*P. aeruginosa*	Piperacillin-tazobactam, IV, 21 days	127 days, ciprofloxacin	No	Unknown	No relapse, no reinfection. Secondary infection unknown.	Yes (not infection-related)
Verkaik et al. 2022 [16]	n = 4 Swab cultures	*P. mirabilis* (1) *S. marcescens* (1) *K. pneumoniae* (1) *P. aeruginosa* (1)	Cefuroxime IV or piperacillin/tazobactam IV (additional vancomycin/gentamicin if indicated) 42 days	No	Surgical driveline relocation performed for infection control in two patients (*Pseudomonas* and *Serratia*)	Unknown	No relapse or reinfection. Secondary infection unknown.	Unknown
Pump/Cannula infection								
Manahan et al. 2006 [18]	Wound culture	*P. aeruginosa*	Broad spectrum antibiotics tailored to imipenem, azithromycin, fluconazole, IV After surgical interventions antibiotics, IV, many weeks	No	Performed for infection control: ePTFE mesh removal; pump-pocket debridement and irrigation; rectus muscle-flap closure; postoperative antibiotic irrigation	61 (surgical interventions until death)	No relapse, no reinfection, no secondary infection.	Yes (ventricular arrhythmias: infection-related)
Manahan et al. 2006 [18]	Wound culture	*P. aeruginosa*	Broad spectrum antibiotics IV and via large-bore drainage catheters Gentamicin and ampicillin-sulbactam after wound cultures tested positive	No	Performed for infection control: wound debridement with omental-flap coverage of exposed foreign material	54 (resolution of infection by antibiotics and surgical interventions until death)	No relapse, no reinfection, no secondary infection.	Yes (VAD removal because of mediastinal bleeds: infection-related)
Cholevas et al. 2026 [30]	PET-CT; CT-guided abscess sampling; FISHseq/16S rRNA gene sequencing; serology	*Coxiella burnetii*	Doxycycline + hydroxychloroquine, PO, 5 months; after HTx switched to ciprofloxacin + doxycycline, PO	Yes, long-term suppressive therapy after HTx	Heart transplantation with LVAD explantation performed for infection control	1095	No relapse, no reinfection, no secondary infection during 3-year follow-up.	No

Abbreviations: ePTFE, expanded polytetrafluoroethylene; HTx, heart transplantation; IV, intravenous; LVAD, left ventricular assist device; PET-CT, positron emission tomography-computed tomography; PO, oral administration.

#### 3.4.2 Pump/Cannula Infections

Two studies reported three cases of Gram-negative pump/cannula infection, caused by *Pseudomonas aeruginosa* (2/3, 66.7%) and *Coxiella burnetii* (1/3, 33.3%). All three patients required surgical source control, and one received suppressive therapy (1/3, 33.3%). The two *Pseudomonas aeruginosa* cases underwent debridement with removal of infected material and flap reconstruction. In the *Coxiella burnetii* case, definitive source control was achieved by LVAD explantation at heart transplantation, followed by long-term suppressive antimicrobial therapy without recurrence during three years of follow-up.

### 3.5 Bloodstream Infections

Six studies reported six cases of Gram-negative device-specific bloodstream infection (Table 5, Ref. [17,21,24,26,28,29]). The most frequently identified pathogen was *Pseudomonas aeruginosa* (4/6, 66.7%), including one MDR isolate. *Cardiobacterium valvarum* (1/6, 16.7%) and MDR *Achromobacter xylosoxidans* (1/6, 16.7%) were also reported. The median duration of antibiotic therapy was 42 days (IQR, 21–51; range, 14–67 days), and suppressive therapy was administered in 3/5 cases (60.0%). Among five cases with available source-control data, one patient underwent heart transplantation for infection control. A second patient underwent heart transplantation after infection resolution and not for infection control. The infection-related mortality rate was 3/6 (50.0%), while 3/6 (50.0%) experienced relapse; one patient died before relapse or reinfection could occur.

**Table 5.** **Characteristics and outcomes of patients with device-specific bloodstream infections**.

Reference	Diagnostics	Infected site	Causative bacteria	Therapeutic antibiotics (for LVAD infection)	Suppressive therapy afterwards	Surgical source control and heart transplantation	Infection free period/follow-up duration from first infection (in days)	Relapse/reinfection/secondary infection	Death during follow-up (cause)
Simon et al. 2005 [17]	Cultures of exit site drainage. Histopathology during autopsy	LVAD-endocarditis	*P. aeruginosa*	42 days	Unknown	Unknown	Unknown	*Pseudomonas aeruginosa* BSI relapse. Reinfection and secondary infection unknown.	Yes (BSI relapse: infection-related)
Hill et al. 2014 [21]	Ultrasonography, blood culture	Deep driveline, blood stream	*P. aeruginosa*	Ceftazidime, 14 days	Ciprofloxacin, PO	No	0	Multiple relapses and reinfections of BSI with *P. aeruginosa* (304 days from the start of the primary infection until death). No secondary infection.	Yes (intracranial mycotic aneurysm: infection-related)
Heil et al. 2015 [24]	Blood culture, a PET scan was done to confirm the LVAD infection	LVAD, blood stream	*P. aeruginosa*	Cefepim, 26 days Meropenem + amikacin, 7 days Meropenem, 21 days Cefepim + colistimethate and later + amikacin	No	No	0	No relapse, no reinfection, no secondary infection.	Yes, 99 days after infection onset (due to primary infection: infection-related)
Peghin et al. 2018 [26]	Blood cultures and moderate 18 F-fluorodeoxyglucose positron emission tomography/CT uptake in the left ventricular apex	LVAD, Blood stream	*P. aeruginosa *(MDR)	Ceftolozane/tazobactam + amikacin for 42 days	No	Heart transplantation performed for infection control	30 days	Relapse with *P. aeruginosa*. No reinfection or secondary infection.	No
Bodro et al. 2021 [28]	Blood culture, PET scan showed no foci	Suspected LVAD and blood stream	*Achromobacter xylosoxidans* (MDR)	Piperacillin-tazobactam, 5 days Tigecycline + colistin, IV, 20 days Cefiderocol + Piperacillin/tazobactam + tigecycline, 14 days Piperacillin/tazobactam + tigecycline, 28 days	Minocycline, PO	No	365 (until end of follow-up)	No relapse, reinfection, or secondary infection during 365-day follow-up.	No during 365-day follow-up
Kumar et al. 2021 [29]	Blood cultures, TTE and thorax and abdomen CT showed no significant findings	Driveline exit site and bloodstream	*Cardiobacterium valvarum*	Ertapenem, 14 days (Prior empiric treatment with piperacillin-tazobactam, IV, plus vancomycin, IV)	Cephalexin 500 mg twice daily, PO, for 348 days until HTx	Heart transplantation performed after infection resolution and not for infection control	348 (last positive culture until HTx)	No relapse, reinfection or secondary infection.	No

Abbreviations: BSI, bloodstream infection; CT, computed tomography; FU, follow-up; HTx, heart transplantation; IV, intravenous; LVAD, left ventricular assist device; MDR, multidrug-resistant; PET-CT, positron emission tomography-computed tomography; TTE, transthoracic echocardiography.

## 4. Discussion

LVAD infections are associated with high recurrence rates and significant clinical burdens, making the optimization of treatment strategies a critical challenge. While guidelines acknowledge that treatment duration and antibiotic choice depend on factors such as infection severity and pathogen type, they also recognize the need for general treatment concepts to assist clinicians [8]. The absence of standardized recommendations contributes to significant variability in clinical practice [6,12,31]. Currently, specific guidance for Gram-negative bacterial LVAD infections is lacking, and existing recommendations rely primarily on expert opinion and evidence extrapolated from other infectious diseases [7,8,9]. To address this gap, we conducted a systematic review to evaluate antibiotic regimens for Gram-negative bacterial LVAD infections and their clinical outcomes, aiming to provide general treatment concepts.

Our findings highlight the scarcity of robust evidence regarding the treatment of Gram-negative LVAD infections. Most included studies were case reports or small case series, with considerable variation in antibiotic choice, treatment duration, and reported outcomes. Superficial driveline infections were treated with a median antibiotic course of 14 days (IQR, 14–21.5; range, 7–49), whereas deep driveline/pocket infections were treated for a median of 42 days (IQR, 31.5–42; range, 14–42). Pump/cannula infections were infrequently documented but generally required prolonged broad-spectrum antibiotics and surgical source control. Bloodstream infections were treated for a median of 42 days (IQR, 21–51; range, 14–67). These findings are consistent with contemporary reports of deep device infection and *P. aeruginosa* VAD infection, in which prolonged therapy and repeated source-control procedures were frequently required [12,13]. The reported median antibiotic durations may reflect selective reporting rather than evidence supporting an optimal treatment duration. The variability in regimens and limited case numbers precluded identification of optimal antibiotic regimens for Gram-negative LVAD infections.

The identified Gram-negative pathogens were microbiologically heterogeneous, with important differences in virulence, antimicrobial resistance, biofilm formation, and treatment options [32,33,34,35]. Specific resistance mechanisms among Enterobacterales, including extended-spectrum beta-lactamase, AmpC beta-lactamase, or carbapenemase production, were not reported in the included cases, and the limited number of cases prevented pathogen-specific treatment analyses. Nevertheless, restricting the synthesis to Gram-negative infections and stratifying pathogens into broader microbiological groups provides greater specificity than recent LVAD studies that report infections across mixed pathogen groups [36,37].

The scarcity of patient-level evidence likely reflects the small numbers and microbiological heterogeneity of Gram-negative LVAD infections within individual centers, limiting reports largely to isolated cases or small series. Building on these findings, we intend to initiate a multicenter study using Research Electronic Data Capture (REDCap), a secure web-based platform for standardized collection of patient-level treatment strategies and outcomes across LVAD centers. In parallel, further research into Gram-negative biofilm formation, antimicrobial penetration, and anti-biofilm device strategies is needed to improve treatment and prevention.

Management of LVAD-specific infection generally combines pathogen-directed therapeutic antibiotics with surgical source control and/or suppressive antimicrobial therapy when indicated [7,8]. We descriptively summarized their use and outcomes, including the type and timing of surgery and the indication and duration of suppressive therapy where reported. However, the limited and heterogeneous evidence precluded reliable conclusions regarding their optimal indications, effectiveness, advantages, and disadvantages.

## 5. Limitations

This review provides a comprehensive assessment of Gram-negative bacterial LVAD infections, an area with limited dedicated research, and highlights critical knowledge gaps. In addition, it compares reported treatment practices with guideline recommendations, illustrating the lack of standardization in clinical management.

However, several limitations should be noted. The small number of cases limits the ability to draw definitive conclusions or provide clear recommendations and underscores the need for further research. Many included studies were case reports, which tend to focus on challenging or refractory infections and may introduce publication bias. Variability in study design, treatment regimens, and follow-up duration further limited our ability to derive clear recommendations. Heterogeneous and sometimes incomplete follow-up may have resulted in underascertainment of relapse, reinfection, and subsequent management. Patients who survived longer had more opportunity for these outcomes to be observed, potentially introducing survivorship-related bias. In addition, early death, heart transplantation, and LVAD explantation may have acted as competing events by precluding the subsequent observation of recurrence, suppressive therapy, or surgery. Recurrence, subsequent interventions, and infection-related mortality should therefore be interpreted together when assessing treatment success. As with any systematic review, the possibility that eligible studies were missed cannot be completely excluded. Given the limited evidence base, even a small number of missed studies could influence the reported treatment durations and outcome proportions; however, the broad screening strategy, independent assessment by two reviewers, and manual reference-list screening reduced this risk. Restriction to English-language publications may also have introduced language bias, although only seven non-English full-text articles were excluded and its impact is therefore expected to be limited. Furthermore, the review was not prospectively registered and no formal protocol was prepared, which may increase the risk of reporting bias.

## 6. Conclusions

The treatment of Gram-negative bacterial LVAD-specific infections remains highly variable, with significant differences in antibiotic regimens, duration, and clinical outcomes. To enhance the management of Gram-negative LVAD infections, robust studies are needed to establish standardized antibiotic regimens tailored to infection type and pathogen. Until such robust data are available, treatment decisions will remain individualized, and clinical uncertainty will persist, emphasizing the critical need for further research. We encourage clinicians to publish detailed data on antibiotic treatment for Gram-negative LVAD infections, including successful cases, to enhance our understanding of the most effective treatment strategies for LVAD-specific infections, which remain prevalent and associated with significant morbidity.

## Data Availability

Not applicable.
