# Supplementary material for: Antibiotic Treatment of Gram-Negative Bacterial Left Ventricular Assist Device Infections: A Systematic Review
Source: Rev Cardiovasc Med. 2026 Oct 15;27(10):52885. doi: 10.31083/RCM52885 (PMC13732660; doi:10.31083/RCM52885)
Supplement: Supplementary file 1 [file 2153-8174-27-10-52885-s1.zip › Supplementary Material/Supplementary Material.docx]

**Supplementary Material**

**Antibiotic treatment of gram-negative bacterial left ventricular assist device infections: a systematic review**

**Supplementary Table 1. Complete database-specific search strategies**

Final search date: April 6, 2026. Complete Boolean operators, field codes, controlled vocabulary terms, free-text terms, and database-specific syntax are provided below. No record counts are repeated here because study identification and deduplication are presented in Figure 1 of the main manuscript.

| **Database** | **Platform/approach** | **Search strategy** |
| --- | --- | --- |
| Embase | Embase.com | ('ventricular assist device'/de OR 'left ventricular assist device'/exp OR (LVAD OR LV-assist-device* OR ventricular-assist-device* OR HeartMate* OR HeartWare*):ab,ti,kw) AND ('Gram negative bacterium'/exp OR (Enterobacter* OR Klebsiella* OR Pseudomona* OR Acinetobact* OR Proteus OR Serratia OR Escherichia-coli OR e-coli OR Gram-negative*):ab,ti,kw) AND [2005-2030]/py |
| MEDLINE ALL | Ovid | (Heart-Assist Devices/ OR (LVAD OR LV-assist-device* OR ventricular-assist-device* OR HeartMate* OR HeartWare*).ab,ti,kf.) AND (exp Gram-Negative Bacteria/ OR (Enterobacter* OR Klebsiella* OR Pseudomona* OR Acinetobact* OR Proteus OR Serratia OR Escherichia-coli OR e-coli OR Gram-negative*).ab,ti,kf.) AND 2005:2030.(sa_year). |
| Web of Science Core Collection | Web of Knowledge | TS=(((LVAD OR LV-assist-device* OR ventricular-assist-device* OR HeartMate* OR HeartWare*)) AND ((Enterobacter* OR Klebsiella* OR Pseudomona* OR Acinetobact* OR Proteus OR Serratia OR Escherichia-coli OR e-coli OR Gram-negative*))) AND PY=2005-2030 |
| Cochrane Central Register of Controlled Trials | Wiley | ((LVAD OR LV NEXT assist NEXT device* OR ventricular NEXT assist NEXT device* OR HeartMate* OR HeartWare*):ab,ti,kw) AND ((Enterobacter* OR Klebsiella* OR Pseudomona* OR Acinetobact* OR Proteus OR Serratia OR Escherichia NEXT coli OR e NEXT coli OR Gram NEXT negative*):ab,ti,kw) |
| Google Scholar | Publish or Perish; first 50 top-ranked results | LVAD\|'left ventricular assist device'\|HeartMate\|HeartWare 'gram negative'\|Enterobacter\|Enterobacterales\|Enterobacteria\|Klebsiella\|Pseudomonas\|Acinetobacter\|Proteus\|Serratia\|Escherichia\|'E coli' |

Note: Google Scholar results were downloaded to EndNote using Publish or Perish. No database limits other than those explicitly shown in the search strategies were applied.

**Supplementary Table 2. Data availability and reasons for missingness for the variables reported in Table 2 among the 25 included cases**

| **Variable** | **Cases with available data, n/N (%)** | **Cases with missing data, n/N (%)** | **Reason for missingness** |
| --- | --- | --- | --- |
| Antibiotic treatment duration | 19/25 (76.0%) | 6/25 (24.0%) | An exact treatment duration was not reported for two superficial driveline/exit-site infections, one deep driveline/pocket infection, and two pump/cannula infections. For one bloodstream infection, the duration of the final antibiotic regimen was not reported. |
| Surgical source control | 21/25 (84.0%) | 4/25 (16.0%) | Source-control status could not be determined for two cases from Simon et al. [17] and for two superficial driveline/exit-site cases in which heart transplantation occurred but the indication was not reported. |
| Suppressive therapy | 24/25 (96.0%) | 1/25 (4.0%) | Suppressive therapy status could not be determined for one bloodstream infection case from Simon et al. [17]. |
| Relapse | 25/25 (100.0%) | 0/25 (0.0%) | No missing data. |
| Reinfection | 23/25 (92.0%) | 2/25 (8.0%) | For two cases from Simon et al. [17], patient-level follow-up and the timing of subsequent infections were insufficiently reported to determine whether reinfection occurred according to the predefined >6-month criterion. |
| All-cause mortality | 20/25 (80.0%) | 5/25 (20.0%) | For one case from Simon et al. [17], individual vital status could not be determined. For four cases from Verkaik et al. [16], mortality was not reported at the individual-patient level. |
| Infection-related mortality | 20/25 (80.0%) | 5/25 (20.0%) | For the same five cases, infection-related mortality could not be determined because individual vital status and cause-of-death data were unavailable. |

Note: Data were considered missing when the respective variable could not be determined at the individual-patient level from the source publication. Exact antibiotic treatment duration was considered available only when a numerical duration could be established.

**Supplementary Fig. 1.** Distribution of Gram-negative pathogens identified in LVAD-specific infections. Pathogens are ordered from highest to lowest frequency. Data are presented as the absolute number and percentage of all 28 identified pathogens, n (%).
